# Biological markers for non-celiac gluten sensitivity: a question awaiting for a convincing answer 

**Published:** 2018

**Authors:** Enzo Ierardi, Giuseppe Losurdo, Domenico Piscitelli, Floriana Giorgio, Annacinzia Amoruso, Andrea Iannone, Mariabeatrice Principi, Alfredo Di Leo

**Affiliations:** 1 *Section of Gastroenterology, Department of Emergency and Organ Transplantation, University of Bari “Aldo Moro”, Italy*; 2 *Section of Pathology, Department of Emergency and Organ Transplantation, University of Bari “Aldo Moro”, Italy*

**Keywords:** Gluten sensitivity, Marker, Diagnosis, Cytokines, Immunohistochemistry

## Abstract

Non Celiac Gluten Sensitivity (NCGS) is characterized by immunological, morphological or symptomatic manifestations precipitated by gluten ingestion in individuals without celiac disease (CD). The most important challenge in NCGS is the diagnosis, currently based only on clinical observation. The “Salerno criteria” have been pointed out to achieve a reliable diagnosis even if they lack immediacy and practicality, thus making questionable patient’s adherence. Therefore, biological indicators supporting the clinical diagnosis of NCGS are advisable. For these reasons, many attempts have been performed in order to identify possible serological, immunological, histopathological, immunohistochemical and pathophysiological aspects characterizing this condition with the aim of using them for diagnostic purposes. In the present narrative review, we carried out an update of the current scenario of potential markers of NCGS. The main fault of available studies is that, in most cases investigations have been pointed out towards molecules, which cannot be searched in the current laboratories of clinical analysis. Therefore, the matter has been confined within basic research. Additionally, in these studies, sensitivity and specificity of biological markers were not computable. This is a relevant limit, since an ideal test for NCGS should have a good discriminative power against both CD and other causes of microscopic enteritis. Until now, serological tests have failed, while the search for a soluble marker indicative of activation of innate immune system as well as immunohistochemistry could be the promising bases for the development of appropriate investigations in the future.

## Introduction

 The concept of Non Celiac Gluten Sensitivity (NCGS) has been developing in recent years until the recognition of this condition as a gluten related disease with peculiar characteristics (1). According to the Oslo definition (2), it consists of “a variety of immunological, morphological, or symptomatic manifestations that are precipitated by the ingestion of gluten in individuals in whom celiac disease has been excluded”. Gastrointestinal manifestations, such as diarrhea, abdominal pain and bloating are the dominant features. However, NCGS may display headache, fatigue, osteopenia or skin manifestations. NCGS shows peculiar clinical features in comparison to celiac disease (CD), such as the onset of symptoms shortly after wheat ingestion followed by a prompt response to gluten free diet. Simultaneously, villous atrophy and serological markers of CD are absent. The pathogenesis of NCGS is almost obscure. A selective involvement of innate immunity has been invoked (3) as well as an immune response to wheat constituents other than gluten (amylase-trypsin inhibitors) has been hypothesized (4).

**Figure 1:**
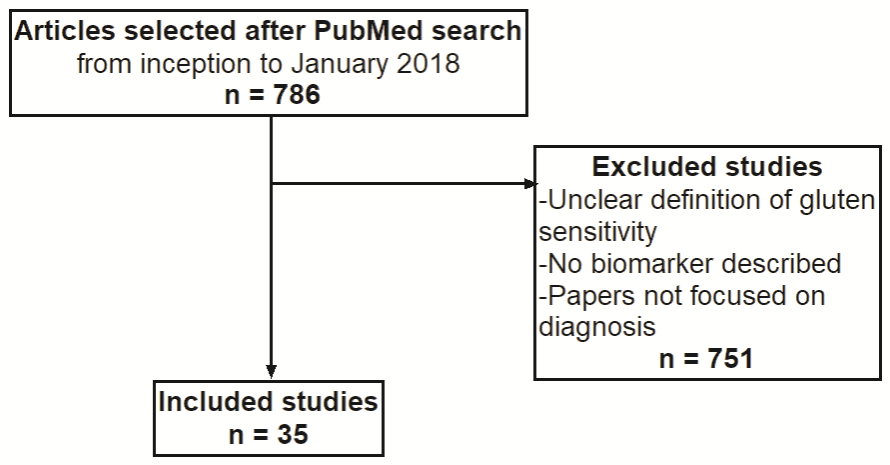
Flowchart summarizing the process of Pubmed search and articles selection.

The most important challenge in NCGS is the diagnosis. Currently, the diagnosis is based only on clinical observation and relies on the exclusion of CD and other possible causes of diarrhea and abdominal pain. In this setting, a subjective complaint of a relationship between symptoms and gluten ingestion, which in several reports reaches a prevalence of 10.6%, may be a confounding factor (5). It is evident that this value cannot be a realistic percentage and is presumably due to the so-called “nocebo effect”. On these bases, the “Salerno criteria” have been pointed out in order to achieve a more solid diagnosis (6). Accordingly, diagnostic protocol encloses two steps: an initial evaluation of a gluten free diet followed by a double blind placebo controlled challenge with crossover. During this phase, symptom evaluation is guided by a Gastrointestinal Symptom Rating Scale filled out by patients. This protocol has been demonstrated to reach a high level of evidence for diagnosing NCGS. For example, it has been shown that only the 14% of self-reported gluten sensitivity is really suffering from this condition (7). Nevertheless, the whole procedure requires nine weeks and, for this reason, it lacks immediacy and practicality and patient adherence may be questionable. Therefore, biological indicators supporting the clinical diagnosis of NCGS are advisable. Consequently, the aim of the present narrative review was to give an updated scenario of potential markers of NCGS. For this reason, we entered in the PubMed database the following key words: non celiac gluten sensitivity, biomarker, serology, anti-gliadin antibodies, intestinal permeability, toll like receptors, cytokine, interleukin, immunohistochemistry, intraepithelial lymphocytes and diagnosis. Since the literature data were extremely heterogeneous, we were unable to perform a systematic review, therefore we planned to carry out a narrative review. The selection process of the papers selected for this review is summarized in Figure 1.

**Serology**

As above reported, NCGS differs from CD for the absence of typical auto-antibodies, such as anti-transglutaminase or anti-endomysium. Nevertheless, some reports showed that about half of the patients with NCGS experienced positivity for native IgG anti-gliadin antibodies. At this regard, Volta *et al.* demonstrated in 2012 a value of prevalence of the 56.4% of positive IgG anti-gliadin (8) and comparable percentages were confirmed by successive cohort studies (9, 10). Interestingly, the adherence to gluten free diet was demonstrated to allow negativity of such antibodies in the 93.2% of cases (11). Finally, even in pediatric age a noticeable positivity for IgG anti-gliadin antibodies (66%) was observed (12). 

Additionally, objective markers of systemic immune activation and gut epithelial cell damage in individuals with sensitivity to wheat in the absence of CD have been demonstrated (13). In detail, these markers are represented by increased levels of soluble CD14, lipopolysaccharide-binding protein and antibody reactivity to microbial antigens, indicating systemic immune activation (IgM anti-Endotoxin Core, which is a hint of B cell response towards microbial antigens). These findings may encourage additional researches aimed to assess potential biological indicators of NCGS. 

**Cytokines and Immunology**

The investigation of soluble mediators of inflammation, in particular cytokines (chemokines and interleukines – IL) and immune system cells constitute a relevant field of research about NCGS. Indeed, there is strong evidence that the involvement of innate immune system is essential in NCGS, while acquired immunity has a negligible value. This aspect has been invoked to explain the well-known negativity of CD serological markers in this condition. 

Therefore, several studies have been pointed out on the evaluations of possible markers of native immunity in NCGS. Two basic studies from Sapone *et al.* (14, 15) dearly supported this hypothesis by showing an increase of mRNA codifying for toll like receptors (TLR)-2 and claudin 4. TLRs are receptors activated by nonself-antigens during innate immune response. Claudin 4 is a member of a family of molecules which are integral components of tight junctions. 

Nevertheless, such results have not been replicated in successive studies. Picarelli *et al.* (16) found that immunohistochemical expression of TLR2 in duodenal mucosa of NCGS was comparable to that of CD and controls. In our experience, mRNA-TLR2 had similar levels in subjects with CD and controls and their expression failed to predict NCGS development in a cohort of patients with microscopic enteritis (17). Additionally, a satisfactory predictive performance was observed for interferon gamma and tissue transglutaminase 2 mucosal expression. 

Recently, the levels of CD14 lymphocytes and lipopolisaccharide binding protein have been shown to be higher in NCGS compared to healthy controls and CD (13). These biological markers are indicators of innate immune activation against bacterial antigens, thus confirming the hypothesized original pathogenetic pathway of this condition. This finding did not agree with the experience of Di Sabatino *et al*. (18), who failed to find differences in the supernatants of *ex vivo* cultured duodenal samples of patients with self-reported NCGS in the expression of cytokines characterizing innate (IL6, IL1, IL23, IL27, IL32 and tumor necrosis factor alpha) and adaptive immunity (IL4, IL5, IL10, IL13, IL17A, interferon gamma). However, this study has the remarkable limit due to the enrollment of patients with self-reported gluten sensitivity.

Another approach to cytokine analysis in NCGS has been based on the provocative stimulus of gluten on immune cells both *in vivo* and *in vitro* and interesting, even if preliminary, data have been obtained. A gluten challenge in patients with NCGS induced an increase in intraepithelial lymphocytes (IELs) and mucosal interferon gamma, while no change in heat shock protein 27 and 70 was recorded (19). Similarly, gliadin was not able to upregulate some markers of basophil activation such as CD63 and CD203c in duodenal biopsy cultures (20). Finally, the exposure to gluten induced the secretion of CXCL10 chemokine by peripheral mononuclear blood cells of patients with NCGS (21, 22).

**Table 1:** Advantages and disadvantages of currently available diagnostic potential investigations for non celiac gluten sensitivity

Diagnostic method	Advantages	Disadvantages
Serology	The test is easily available in laboratories Data from literature show high agreement Low diagnostic power (positivity in only the 50%)	
Cytokine assay	Highly standardized technique High specificity Conflicting result Low sensitivity Available only for research purposes	
Histopathology and immunohistochemistry	The test is easily available in laboratories Excellent sensitivity and specificity Very few studies in literature	
Permeability test	Highly standardized technique Low sensitivity Conflicting results	
Double blind placebo controlled oral provocation test (Salerno criteria)	High consensus level High sensitivity and specificity Long duration time	

**Histopathology and Immunohistochemistry**

NCGS is characterized by a normal duodenal histological picture (Marsh 0 stage) as well as by an increase of IELs above 25/100 enterocytes with a normal villous architecture (Marsh 1 stage). Therefore, NCGS could be acknowledged as a cause of duodenal lymphocytosis/microscopic enteritis (23, 24). Since the first reports of NCGS, CD3 duodenal staining for IELs showed that this condition exhibited intermediate count values between controls and CD (14). Furthermore, we found that duodenal lymphocytosis of 15-25 IELs/100 enterocytes may be a risk factor for the evolution towards NCGS in a group of patients with microscopic enteritis, with an odds ratio of 28.59 (25). Villanacci *et al.* suggested that a linear T-lymphocyte infiltration (hallmarked by CD3 staining) in the lamina propria could characterize NCGS (26). However, this finding was observed in a single-center experience and involved about the 78.5% of the patients. Eosinophilia in the lamina propria has also been proposed as a possible peculiar NCGS feature (10, 16). Our latest study demonstrated that the immunohistochemistry for CD4 (a marker of T-helper lymphocytes) in the lamina propria had a sensitivity of 100% and a specificity of 90% to discriminate between NCGS and CD as well as a sensitivity of 87.5% and a specificity of 85% in differentiating between healthy controls and NCGS (27). Indeed, CD8+ cell infiltration in the lamina propria of NCGS was intermediate between controls and CD. Moreover, CD117 staining allowed detecting higher levels of positive cells in NCGS than in controls and CD. In this case, a value of 134 cells/mm^2^ discriminated between NCGS versus CD and controls with a sensitivity of 75% and a specificity of 55% (27). Since CD117 is a staining which could identify mast cells, this finding could support the results of some reports claiming that an allergic sensitivity to food allergens other than wheat may play a role in NCGS (28, 29). At this regard, however, it should be highlighted that mast cells are directly involved in all gluten related disorders, since gliadin is able to induce their degranulation with a consequent inflammatory cytokine production (30). 

Finally, a very interesting finding was revealed by the study of rectal biopsy samples of patients with NCGS. Indeed, an increase in interferon gamma producing type-1 innate lymphoid cells with a characteristic CD45(+) T-bet(+), CD56(-), NKP44(-), and CD117(-) pattern was observed. Of interest, this aspect was reverted by a gluten free diet (31).

**Pathophysiology**

The determination of intestinal permeability by means of lactulose/mannitol test has been poorly investigated in NCGS. This investigation involves the administration of a solution containing lactulose and mannitol; an enhanced permeability is indicated by an increase in lactulose and a decrease in mannitol absorption (32). An experience from Sapone *et al*. performed in 26 NCGS subjects, 42 CD patients and 39 healthy gluten tolerant controls, failed to demonstrate an alteration of intestinal permeability in NCGS (14). On the other hand, further evidences showed opposite results. Hollon *et al.* (33) measured permeability by transepithelial electrical resistance in cultured duodenal biopsy samples and displayed that gliadin increased intestinal permeability in NCGS more than in CD patients and healthy subjects. Furthermore, fatty acid binding protein 2, a surrogate marker of epithelial damage and permeability, was found higher in NCGS than in controls (13). Therefore, the debate about intestinal permeability in NCGS is still open, since there are heterogeneous data.

Finally, oral gluten provocation test has been tested in a single experience (16). Such investigation consists in the application of gluten containing patches on the oral mucosa. The test is positive if mucosal hyperemia, edema, blisters or burning occur in the mouth. The test was positive in the 75% of NCGS, statistically higher than CD both under gluten containing (15%) and free diet (25%). The test did not give a positive result in healthy controls. This finding, when confirmed in a multicenter study involving a large population sample, could be a simple and effective test to diagnose NCGS. 

## Conclusion

Despite many attempts and promising preliminary indications, at the moment no biological marker has shown an adequate reliability for the diagnosis of NCGS, which remains based on clinical exclusion criteria. Therefore, clinical suspicion is still fundamental in this field and double blind placebo controlled challenge with crossover remains the diagnostic “gold standard”. However, it shows obvious limitations related to the long duration and the lack of practicality. Therefore, a biological marker for NCGS may be very advantageous. Advantages and disadvantages of currently available diagnostic potential investigations have been summarized in table 1.

The main flaw in the studies aimed to identify a reliable marker is that, in most cases, investigations have been pointed out towards molecules which cannot be searched in the current laboratories of clinical analysis. Therefore, the matter has been confined within basic research. Additionally, in most studies, sensitivity and specificity of biological markers were not computable. This is a relevant limit, since an ideal test for NCGS should have a good discriminative power against both CD and other causes of microscopic enteritis. Until now, serological tests have failed except for native IgG anti-gliadin antibodies, which are present, however, only in the 50% of patients (34, 35). Therefore, the search for a soluble marker indicative of activation of innate immune system as well as immunohistochemistry peculiarities could be the promising bases for the development of appropriate investigations in the future. However, the right road seems to be still long.

## Conflict of interests

The authors declare that they have no conflict of interest.
